# A two decade dementia incidence comparison from the Cognitive Function and Ageing Studies I and II

**DOI:** 10.1038/ncomms11398

**Published:** 2016-04-19

**Authors:** F. E. Matthews, B. C. M. Stephan, L. Robinson, C. Jagger, L. E. Barnes, A. Arthur, C. Brayne, Cognitive Function and Ageing Studies (CFAS) Collaboration, A. Comas-Herrera, R. Wittenberg, T. Dening, C.F.M. McCracken, C. Moody, B. Parry, E. Green, R. Barnes, J. Warwick, L. Gao, A. Mattison, C. Baldwin, S. Harrison, B. Woods, I.G. McKeith, P.G. Ince, S.B. Wharton, G. Forster

**Affiliations:** 1 MRC Biostatistics Unit, Institute of Public Health, Cambridge CB2 0SR, UK; 2 Institute of Health and Society, Faculty of Medicine, Newcastle University, Newcastle NE4 5PL, UK; 3 Department of Public Health and Primary Care, Cambridge Institute of Public Health, Cambridge University, Cambridge CB2 0SR, UK; 4 School of Health Sciences, University of East Anglia, Norwich NR4 7TJ, UK; 5 Personal Social Services Research Unit (PSSRU), London School of Economics and Political Science, London WC2A 2AE, UK; 6 Institute of Mental Health, University of Nottingham, Nottingham NG7 2TU, UK; 7 Institute of Psychology, Health and Society, University of Liverpool, Liverpool L69 3GL, UK; 8 Medical Research Council, London WC2B 4AN, UK; 9 Department Social Science, Health & Medicine, Kings College, London, Strand, London WC2R 2LS, UK; 10 Alzheimer's Society, Devon House, London E1W 1LB, UK; 11 Royal Devon & Exeter Hospital, Exeter EX2 5DW, UK; 12 Dementia Services Development Centre (DSDC Wales), Bangor University, Bangor LL57 2PZ, UK; 13 Newcastle University Institute for Ageing, Newcastle upon Tyne NE4 5PL, UK; 14 Department of Neuroscience, Sheffield Institute for Translational Neuroscience, University of Sheffield, Sheffield S10 2HQ, UK

## Abstract

Dramatic global increases in future numbers of people with dementia have been predicted. No multicentre population-based study powered to detect changes over time has reported dementia incidence. MRC Cognitive Function and Ageing Study (CFAS) undertook baseline interviews in populations aged 65+ years in England and Wales (1989–1994). Three areas (CFAS I) were selected for new sampling two decades later (2008–2011) with same geographical boundaries, sampling and approach methods (CFAS II). At 2 years CFAS I interviewed 5,156 (76% response) with 5,288 interviewed in CFAS II (74% response). Here we report a 20% drop in incidence (95% CI: 0–40%), driven by a reduction in men across all ages above 65. In the UK we estimate 209,600 new dementia cases per year. This study was uniquely designed to test for differences across geography and time. A reduction of age-specific incidence means that the numbers of people estimated to develop dementia in any year has remained relatively stable.

## Abstract (web summary)

Future dramatic rises in dementia are widely reported, assuming no change in incidence. Matthews and colleagues report that, in contrast to such statements, age-specific incidence has dropped over 20 years, with overall incidence of dementia remaining stable in a large multi-site population study from England.

## Graphical abstract

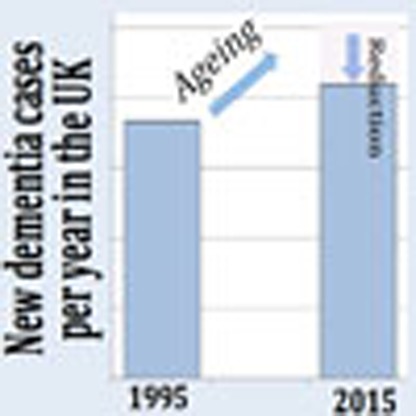

Dementia continues to be a topic of major international interest with successive reports suggesting large increases throughout the world in the next decades^1^,^2^,^3^,^4^. Following governmental concern and the 2013 G8 summit, the World Dementia Council has been established to facilitate greater attention both in research to support reduction of risk, better diagnosis and treatments, and also support for those at risk of, and with, the dementia syndrome. In contrast, new emerging studies provide data that dementia occurrence might be changeable across generations with both decreases and increases reported^5^,^6^,^7^,^8^,^9^. During this time there have been considerable changes in diagnostic practice, and it is a challenge to maintain stability in diagnostic and methodological practice, which themselves could easily drive any changes seen in prevalence and incidence.

Over the last two decades there has been an explosion of interest in intermediate cognition with multiple and changing diagnostic criteria, which now include biological measures^10^. In the context of such rapid change, only studies where identical diagnostic methods are maintained can provide any indication of whether dementia occurrence in populations is truly changing. When there are attempts to take changes into account in meta-analyses of prevalence studies reported changes have been shown to be, to a large extent, accounted for by these design and clinical practice changes^8^. Where there has been an attempt to control for changes in methodology and diagnostic thresholds the general consensus from the small number of studies in the US and Europe suggest lower prevalence at given ages, resulting in, at least in the UK, relatively stability in estimation of overall numbers of prevalent cases^11^. In two of the European studies previous findings of higher risk in women are confirmed, with much, if not all, of the drop in prevalence and incidence being driven by a drop in the men's rates^8^,^12^. A synthesis of European studies suggests that further evidence from dedicated studies is needed to provide definitive evidence on incidence^13^.

Many risk factors for dementia have changed quite dramatically over the last decades both increasing and decreasing in prevalence and severity, with many linked to healthy vascular systems^14^, including diabetes and metabolic syndrome. Education has also been transformed over the years with longer years and greater expectations with associated reports of improved cognition across generations. Although there is limited trial evidence, estimates of the combination of these risk factors' impact on attributable risk suggests that up to 30% might be ‘preventable'^15^. In addition, there is strong and continually emerging evidence of the influence of cognitive, physical and social engagement “protecting” or compensating for existing neuropathology in the brain^16^,^17^,^18^. Cognition itself has been improving within populations^19^,^20^,^21^. Given there have been major changes in many Western and high-income societies in these domains, the occurrence of dementia might change across current generational cohorts.

Prevalence of dementia is the result of both incidence and mortality, with incidence considered to be a more robust comparator measure across time and geography because of potential changes in differential mortality between those with and without dementia and changing mortality in populations. No direct comparison of incidence across time in multiple areas, while maintaining identical methodological approaches, has been conducted in the world to date. Here we report on the first such study.

Incidence across the two decades has dropped by 20% (95% confidence of interval (CI): 0–40%). This drop is driven by a reduction in incidence among men at all ages. These findings suggest that in the UK there are just under 210,000 incident cases per year, 74,000 men and 135,000 women. This study was uniquely designed to test for differences in the prevalence and incidence of dementia across geography and time in diverse areas within a single country. A reduction of age-specific incidence holding diagnostic methods steady means that even in the presence of an ageing population the numbers of people estimated to develop dementia in any year has remained relatively stable, providing evidence that dementia in whole populations can change.

## Results

### Baseline and follow-up numbers and characteristics

**Figure 1:**
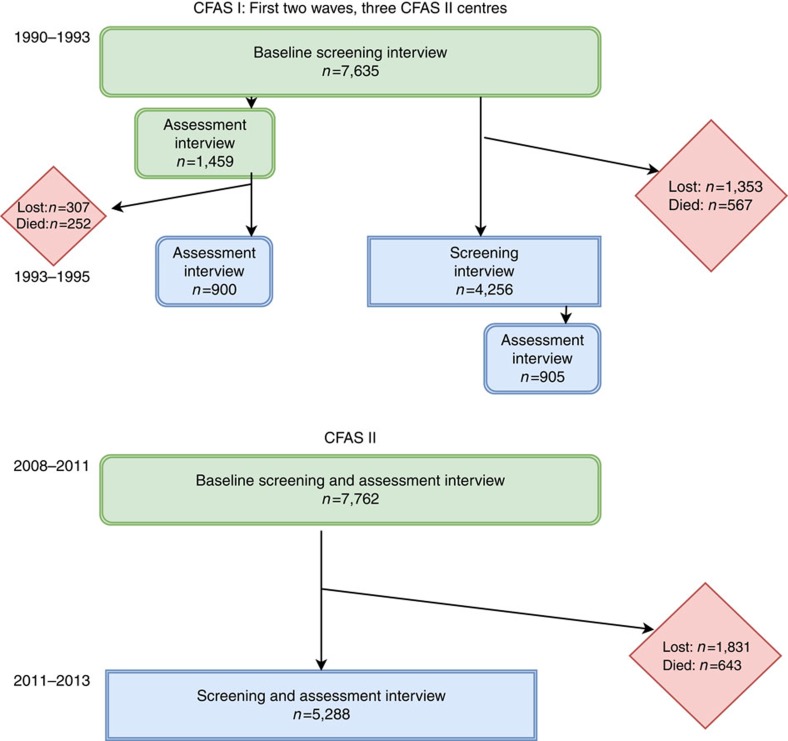
Flow chart of individuals in CFAS I and II. Figure shows flow of 7,635 individuals from CFAS I and 7,762 individuals in CFAS II through the baseline and 2 year follow-up, detailing those who are lost to follow-up and those who died between the waves.

Details of the baseline waves for each study have been presented previously and are not repeated here^11^,^22^. Figure 1 shows the flow of individuals within both the CFAS I and CFAS II studies.

CFAS I: There were 7,635 individuals in the baseline screen, of whom 1,459 took part in the assessment interview. Of these individuals 900 took part in the 2 year follow-up where incidence can be calculated directly. A further 4,256 took part in the re-screen interview of whom 905 took part in the assessment so dementia status at year 2 was seen (with unknown baseline status). In total 1,660 (22%) were lost between waves (1,353 with unknown dementia status at baseline) and there were 819 (11%) deaths (567 with unknown dementia status at baseline). Therefore, response at wave 2 was 76% in those still alive.

CFAS II: There were 7,762 individuals in the baseline and assessment interviews. Of these 5,288 took part in the re-interview, all can be used to calculate incidence directly. A total of 1,831 (24%) were lost between waves and there were 643 deaths (8%). Response rate in those still alive at wave 2 was 74%, very similar to CFAS I. Supplementary table 1 shows the numbers with dementia diagnosis in each study.

### Incidence rates by age and sex

**Figure 2:**
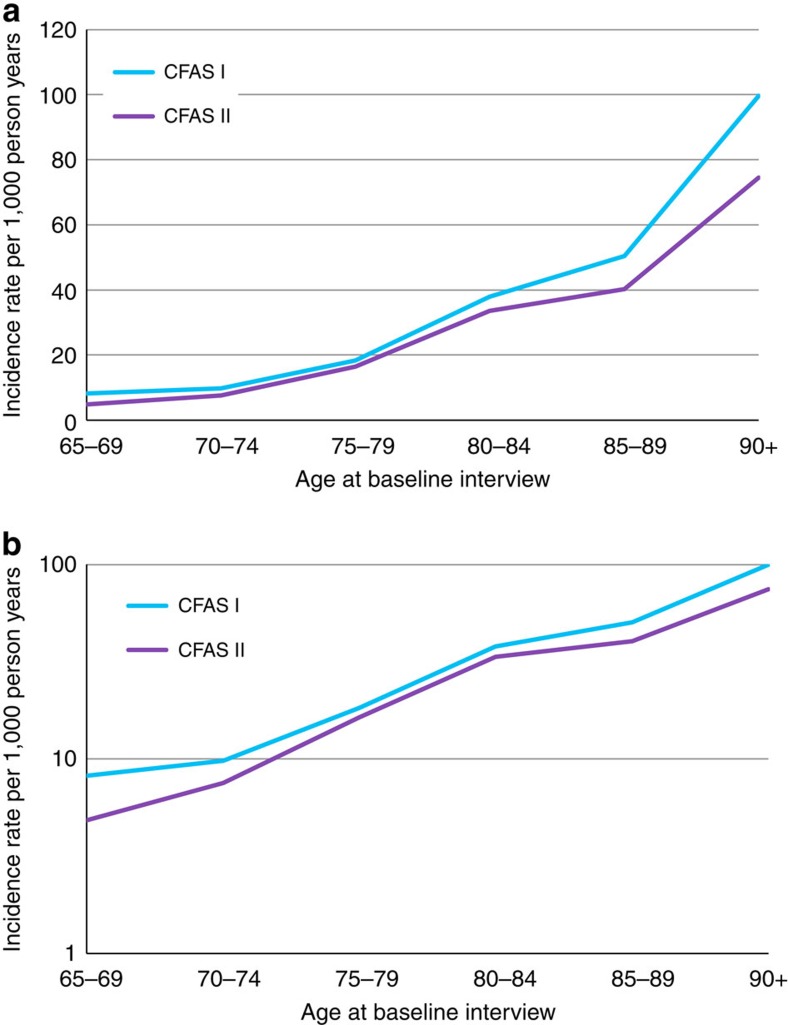
Dementia incidence rates in CFAS I and CFAS II. (**a**) Incidence rate of dementia per 1,000 person years in CFAS I and CFAS II by age at baseline interview. Natural scale. (**b**) Incidence rate of dementia per 1,000 person years in CFAS I and CFAS II by age at baseline interview. Logarithmic scale.

Incidence rates presented are shown in those who were still alive at wave 2 (Table 1). The overall incidence was 20.0 (95% CI: 16.9–23.8) per 1,000 person years in CFAS I and 17.7 (95% CI: 15.2–20.9) in CFAS II. Incidence showed a decrease in most age/sex groups between the two studies. Estimates in CFAS I are less precise due to the sampling design and therefore smaller numbers in the assessment stages. Incidence rates in CFAS I and II for six 5 year age groups are shown in Fig. 2. The reduction in incidence is calculated as an incidence rate ratio (IRR) 0.8 (95% CI: 0.6–1.0, *P*=0.08, Poisson regression, *N*=10,444).

**Figure 3:**
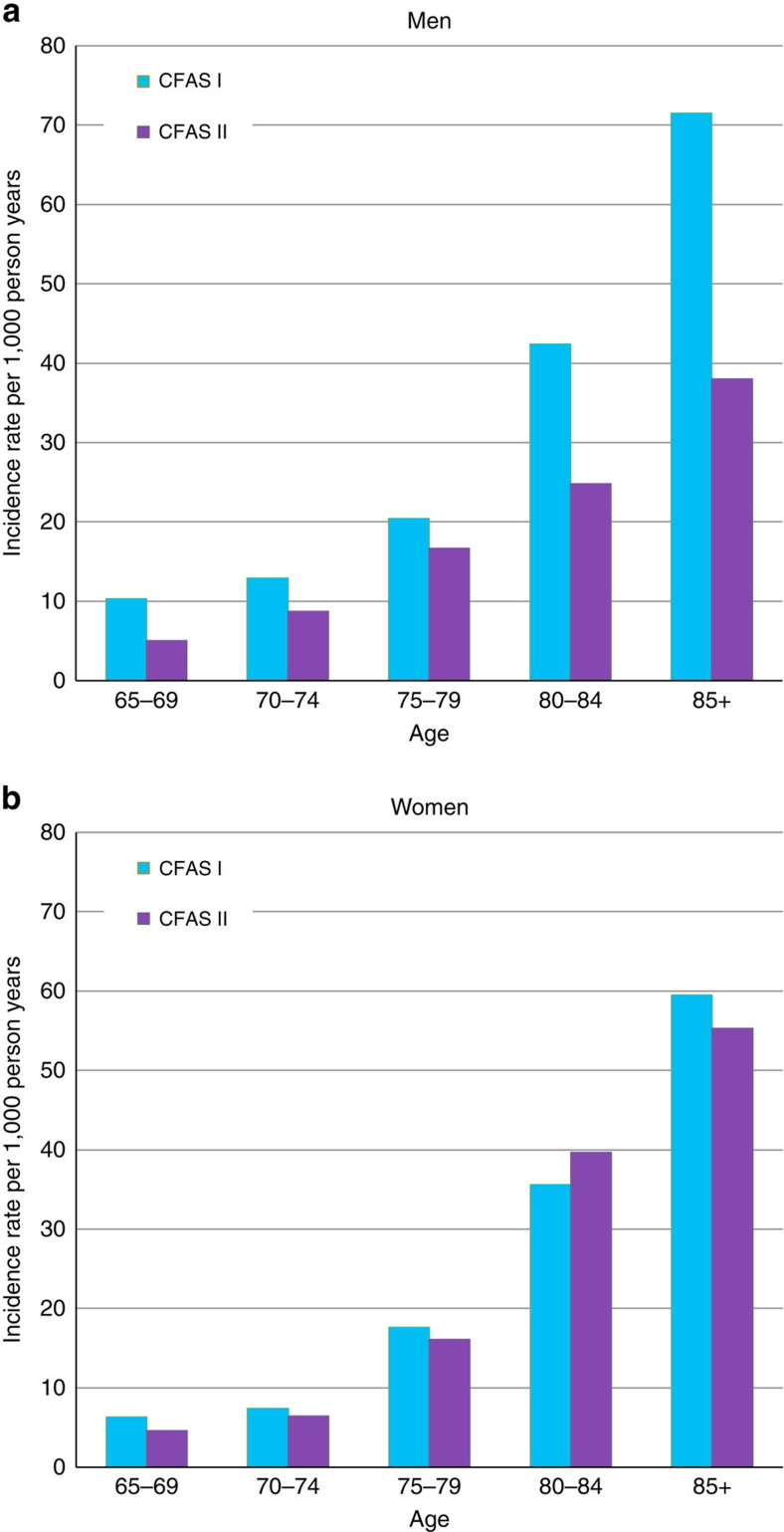
Dementia incidence rates in men and women. (**a**) Incidence rate of dementia per 1,000 person years in men for CFAS I and CFAS II by age at baseline interview. (**b**) Incidence rate of dementia per 1,000 person years in women for CFAS I and CFAS II by age at baseline interview.

There is evidence that the effect is different between men and women (Fig. 3). In CFAS I, there was some evidence that women had lower incidence than men (IRR women:men 0.7 (95% CI: 0.5–1.1, *P*=0.14), not seen in CFAS II (IRR 1.2 (95% CI: 0.9–1.6, *P*=0.23). These effects appear to have been driven by a decrease in the incidence seen in men 0.6 (95% CI: 0.4–0.9, *P*=0.007) but not in women 1.0 (95% CI: 0.7–1.3, *P*=0.9).

### Incidence rates by deprivation and area

Some effect of deprivation is seen in the incidence rates in CFAS II (most versus least IRR 1.5 (95% CI: 1.0–2.2)) but not CFAS I (IRR 1.0 (95% CI: 0.8–1.2), with higher incidence rates in the more deprived (CFAS II trend *P*=0.05), however the effect is attenuated slightly after adjustment for age and sex differences (trend *P*=0.16, IRR 1.3, 95% CI: 0.9–2.0) and further after additional adjustment for area (trend *P*=0.27, IRR 1.3, 95% CI: 0.8–1.9, see Supplementary Table 2), in CFAS I all *P* values >0.4. There is some variation in the unadjusted incidence rates within the three areas, however there is no evidence of a difference in incidence between the three areas (Table 2, all *P* values >0.15 (unadjusted, adjusted for age and sex, and adjusted for age, sex and area).

Adjustment for initial non-response made little difference to the estimates of incidence, as expected, as the effect of the weights primarily apply to individuals with baseline dementia who by definition are excluded in an incidence analysis (non-response adjusted models used).

### Population estimates of incidence cases

**Figure 4:**
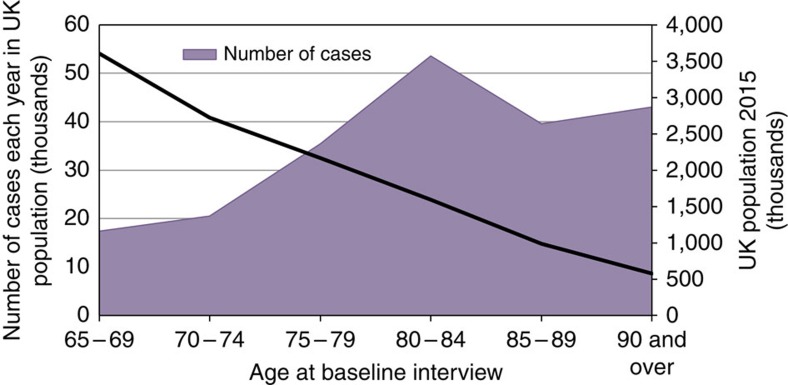
Total number of incident cases in UK in 2015 by age. Total number of new cases of dementia occurring each year by age at baseline interview shown in shaded figure. The line shows the total population in the UK by age group.

On the basis of these models the incidence of dementia in individuals in the UK 1991 was estimated to be 183,000 per year. With no change in the incidence rates, and the known increase in the older population there would be expected to be 251,000 incidence cases per year in the UK in 2015. On the basis of the new estimated incidence rates seen in the population 209,600 people aged 65 and over would be expected to develop dementia according to CFAS II criteria each year. Figure 4 gives the number of incident cases by age group each year in the UK, showing that more than 40,000 incident cases occur every year in individuals aged 80–84, 85–89 and 90 and over despite the decreasing size of the general population at these ages because of mortality.

## Discussion

These findings provide the first multi-area evidence of a drop of 20% in incidence in the population aged 65 and over measured directly, mostly observed in men. Our findings suggest that in the UK there are just under 210,000 incident cases of dementia per year, 74,000 in men and 135,000 in women. This represents a far smaller increase than would have been expected from extrapolation of earlier estimates. Deprivation may well be associated with incidence, though this is more complex to assess than prevalence, due to the relationship between deprivation and survival.

Limitations are unavoidable within such studies. The study is based in three of the original areas, which were chosen to represent the full range of prevalence estimates within MRC CFAS and include urban and rural areas and diversity of geographies. It would have been desirable to conduct the comparison across all areas but this was not fundable. A key strength is that the sampling approach was primary care which in the United Kingdom up to the time of this study was largely on the basis of geography, thus providing excellent opportunity for geographical sampling. The baseline wave of CFAS II had a lower response rate, and in the publication of our prevalence estimates strenuous efforts were made to rule out the possibility that this drove the reduction in prevalence that we observed. The 2 year follow-up had a 74% response rate, almost identical to CFAS I, and the analytical methods address the initial non-response as well as the longitudinal dropout due to refusal in those alive. Other studies have found that incidence is not as affected as prevalence by non-response^23^ and our results did not alter after initial non-response adjustment. The death rate in CFAS I was higher than that seen in CFAS II and hence more individuals are included within the CFAS II analysis despite no change in the longitudinal response rate over time. The baseline interview moved from a two stage design to one stage, and for the 2 year follow-up there were some additions of new measures in the interviews but the CFAS I interviews were represented in CFAS II interviews. CFAS I estimates are less precise due to the earlier two phase design reducing power than CFAS II where all participants received the full screen and assessment content. The interviews were on the basis of standardized methods, which has advantages and disadvantages. The major advantage here is the stability of the interview method and diagnostic approach for study dementia status allocation. In the intervening years the diagnostic criteria have changed many times, with current particular instability due to Diagnostic and Statistical Manual of Mental Disorders (DSM) and International classification of diseases (ICD) refreshment and the increasing popularity of the concept of predementia states^24^. These changes are influencing the way that societies view dementia, risk prediction for dementia and interaction with services. However, the purpose here was to see whether the fundamental stable phenomenon is changing, rather than measuring increases which are likely to be the result of shifting diagnostic boundaries. To test the impact of these societal changes we need to use current extensive phenotyping within the context of such stable methods, and then understand the implications of changing diagnostic boundaries for prediction and prognostication. Previous analysis in our, and other incidence, studies do not generally include ‘interval' dementias in those individuals who die. Here we continue this convention, though future analyses will be undertaken to use information from death certificates to estimate individuals who develop dementia then die quickly between the waves. Our findings show a larger decrease in men than women between the two time periods, but from incidence rates in CFAS I where the rates in women were (up until the oldest old) lower than seen in men. The results of regression to the mean can therefore not be excluded, however, this is potentially unlikely due to the small but consistent decrease seen also in women and due to the large study size and random population sampling.

The new analysis methods give very similar results to the original method for CFAS I. In that investigation England and Wales (MRC CFAS, 5 areas) were estimated to have 163,000 individuals newly meeting dementia criteria each year in 1991 (ref. 25), re-analysis based only on the three CFAS I areas included here is 164,000, and the contemporary analysis for CFAS II applied to current UK population age sex distribution is 170,000, only a 6,000 increase.

Our findings suggest that population brain health is changing, possibly fundamentally, across generations and is likely to be adversely affected by risk factors associated with disadvantage. The positive change seen in Europe may be limited to those countries that have had major investments into population health over the lifecourse of those now in older ages. Such investment has not been experienced across the globe, and the lack of progress in access to education, current patterns of malnutrition in childhood and persistent inequalities within and across countries will play out across the lifecourse of our children and young adults. Within the UK and Europe the reductions we report will be offset within services by the concept of “early” detection, and diagnosis which is driven by a combination of policy initiatives focused on increasing national diagnosis rates^26^, and diagnostic boundary shifts meaning that individuals who were previously not diagnosed with dementia or cognitive impairment are now being tested and referred for specialist assessment with identification of ever milder stages with unknown prognostic significance. Only studies with stable diagnostic methods are able to tease out these secular diagnostic trends from true underlying biological changes.

Incidence rates have indeed declined over the last two decades in these areas, mainly in men with women's rates decreasing less strongly. These provide definitive confirmation of the earlier reports from CFAS II of reduction in prevalence. The finding is consistent across areas. This provides the evidence required using stable diagnostic methods that dementia has gone down within England over the last 20 years.

CFAS II is the first multi-area study globally to have been designed and powered from its outset to compare prevalence and incidence, and test using as identical methods as possible, new independent sampling and steady diagnostic method. The findings now add more robust data to those European studies, which reported on incidence from single sites using indirect measurement. It is also consistent with cohort changes in cognitive profiles upwards^19^,^20^.

It has been long recognized that primary prevention of dementia as opposed to secondary (early detection) or tertiary (mitigation once present) through healthier lifecourse at societal levels, reduced vascular risk and enhanced opportunity for all types of engagement is likely to be more cost effective than national initiatives, such as dementia strategies targeted at earlier and earlier identification of at-risk states^27^, although all approaches should be fully explored for their potential. Our findings support a balancing towards a lifecourse emphasis, although clearly there will always be a need for therapies to target specific disorders or well defined risk groups, and for appropriate care for those whose dementia is in the context of comorbidity and frailty, close to the end of life.

Influential reports in the media and for government continue to promote future scenarios of huge increases of people with dementia in global societies. Undoubtedly this is correct for some areas of the globe. But our study confirms the finding of reduction of not only age-specific prevalence but also incidence in the UK. This provides definitive evidence that dementia in whole populations is changing. Our studies were powered to detect this change and the finding is consistent across the three areas. Policy makers and politicians will need to take into account the now compelling evidence that such changes in the onset and occurrence of dementia are possible. The underlying aetiology may also be changing. Global attention to health across the lifecourse, particularly earlier risk evolution before older age, may be particularly important and remains relatively neglected in societies' search for specific therapies, when in fact, the dementia “tsunami” is in the oldest age groups and characterized by greater complexity than allowed for in current disease models^28^.

## Methods

CFAS I: Between 1989 and 1994 baseline screening interviews in complete population samples of people aged 65 years and over, followed by an assessment interview in 20%, were conducted in five geographical areas of the UK, with a 2 year follow-up (incidence phase). Three of the areas were selected on the basis of representing the range of prevalence estimates (not significantly different between the areas) observed across the areas and to provide rural/urban and north/south areas. These were Cambridgeshire, Newcastle and Nottingham (baseline interviews for these three areas 1991–3).

CFAS I and CFAS II had identical designs, methods and diagnostic approach apart from the simplification of design from two stage to one stage at baseline and incidence phase through combination of screen and assessment interviews, though the CFAS I interview was completely included in CFAS II. Both studies used the UK system of primary care registration, which provides the most robust population sampling frame (including institutions) and allows true geographical sampling held steady over time. Sampling was stratified according to age group (under 75 and 75 and over) to allow sufficient numbers in the older age group. Oversampling from the population register was used to allow for losses (death, incorrect registration, ineligibility and GP refusals, participant or gatekeeper refusal). The primary care practices screened records of patients in selected practices for death and terminal illness^29^.

Fully informed written consent was sought, and when capacity was impaired procedures complied with the UK Mental Capacity Act 2005. CFAS has been approved locally at all centres since its beginning in 1989. After the introduction of multicentre research ethics committees, the study continued to apply for both multicentre and local research ethics committee approval at each centre. Full information on the changes that have occurred over the previous 25 years to the title changes of local research ethics and multicentre ethical committees, along with REC numbers can be found here http://www.cfas.ac.uk/cfas-i/data/#cfasi-ethical-approval.

### CFAS I and II approach

An introductory letter from the general practitioner was followed by a visit from a named study interviewer. Fully informed written consent was sought and when capacity was impaired procedures complied with the UK Mental Capacity Act 2005. At 2 year follow-up all those respondents who had provided an interview and were still alive were re-approached, having first checked with the general practice for terminal illness or other contra-indication to re-approach. Each individual was visited up to three times to maximize response at each time period.

For both CFAS I and II local interviewers were recruited from a range of backgrounds. Some of the interviewers from CFAS I in each area continued to CFAS II across all time points, others were newly recruited. They were provided with identical training by the senior study coordinator who also had continuity from CFAS I. The training consisted of an intensive 1 week course with ongoing practice and training at individual study sites until the interviewer reached a consistent and standardized level.

The CFAS I screening interview included questions on sociodemographic variables, lifestyle, health, activities of daily living (basic and instrumental), cognition, health and social care contact and medication. A sample of 20% was invited for a standardised assessment interview, which included the full Geriatric Mental State examination, containing sufficient information for ascertainment of the dementia syndrome and other neuropsychiatric syndromes in older populations. This provided the background information for the diagnostic algorithm, AGECAT^30^,^31^. For all these interviews an informant interview was also requested. The sample for assessment was a stratified random sample on the basis of Mini Mental State Examination (MMSE) score^32^, AGECAT organicity score and age, with higher selection probabilities at lower cognitive ability. The subset included in the assessment interview were biased towards the cognitively frail (all suspected of having dementia, or who had an MMSE ≤21), together with 1 in 2 to 1 in 10 individuals who were not suspected of having dementia and had MMSE scores above 21. At 2 years in CFAS I those who had not been selected for assessment interview were invited to an incidence screen, with a further 20% invited to assessment (again enhanced for the cognitive impaired). All those who had had assessment at baseline and were willing to be followed up had a combined screen and assessment interview.

In CFAS II this combined screen assessment interview (identical to the two interviews above) was used from the outset, simplifying the design. Overall 20% of respondents were asked to identify an informant—weighted sampling towards the cognitively frail, but also to provide representation of the whole population. This was repeated for the 2 year follow-up.

CFAS I baseline wave was undertaken between 1991–1993 with the incidence wave during 1993–1995. CFAS II was undertaken during 2008–2011 for the baseline wave and 2011–2013 for the incidence wave (see Fig. 1).

The algorithmic approach to diagnosis allows stability over time, without the variability likely to be present whenever those subject to the changes in diagnostic practices over time are involved in diagnosis (whether it be individual clinician or consensus). The GMS-AGECAT has been validated against internationally accepted earlier diagnostic criteria (DSM-IIIR)^33^. Missing data within an interview could prevent an algorithm diagnosis and for individuals with missing data, the same approach was taken for CFAS II as for CFAS I, which was a review of all available information by diagnostician (CB), applying DSM-IIIR criteria. Many of these individuals had severe cognitive impairment and were not able to respond to the interview questions.

Area deprivation tertiles were calculated on the basis of the Townsend deprivation index in 1991 for CFAS I and 2007 (latest available) in CFAS II. Postcodes are used to calculate deprivation for the complete eligible sample (therefore reflecting any non-response deprivation effect).

### Analytical methods

Modelling of dementia incidence has been undertaken using the same methods for both CFAS I and II studies. Investigation of incidence in CFAS II is more straightforward as all individuals were included in the dementia diagnosis if they took part in the interviews (see Fig. 1). In CFAS I a stratified subset of individuals (representing the complete age and cognitive spectrum) at each wave undertook the assessment interviews, so this process needs to be modelled for those missing dementia diagnosis by design.

Full likelihood modelling^25^,^34^ of dementia incidence was undertaken as part of a multiple imputation for non-response using WinBUGS 1.4.3 (ref. 35) and STATA 12 (ref. 36). The full likelihood model used for the living individuals has been presented and discussed further in Clayton *et al*. (ref. 34) and Matthews (ref. 37). It has shown to have sensitivity of 90% and specificity of 95% to real and simulated data. Further extensions of the missing data model were shown to not improve the prediction of the missing data^37^. Individuals who took part in the first wave interview and were still alive at the 2 year follow-up were modelled for dementia prevalence, incidence and the relationship between MMSE (the screening instrument) and dementia status, together with a missing data model for longitudinal loss to follow-up. The model is composed of three parts, prevalence, incidence and missing data. Missing data and screening models included adjustment for deprivation, gender, age and residential setting. Longitudinal missing data additionally included information on dementia and MMSE from the previous wave, together with adjustment for known attrition factors^38^,^39^. Full likelihood models for imputing dementia status at wave 1 and wave 2 consist of four parts of the model:

1. Prevalence of dementia at wave 1—dementia potentially related to age and sex.
2. Incidence of dementia at wave 2—incidence potentially related to age and sex in those who were not demented at wave 1.
3. Relationship between MMSE, missing MMSE and dementia (essential component of CFAS I design)—model includes age, sex, deprivation, care settings, area (study site) and dementia status.
4. Longitudinal missing data—longitudinal missing data is related to age, sex, care status, area (study site), deprivation, disability, dementia at wave 1, MMSE at wave 1 and factors associated with attrition.

Factors associated with attrition were combined from smoking history, education, social class, disability, self-reported health, memory problems and proxy/short interviews using factor analysis to ensure that all were present or absent to reduce the missing data models required during the modelling process. Three unique factors were found in each study.

The model can provide a dementia incidence rate, however initial non-response is not included within the model and hence a two stage process was employed.

All individuals with missing data received an imputed dementia diagnosis from the full likelihood models at wave 1 and wave 2 and person years were also imputed for each individual where a follow-up interview was not undertaken. Individuals with incident dementia at wave 2 (both known and imputed) were assumed to have developed their dementia mid way between the two waves. A total of 100 imputed datasets were calculated. In addition, person time of observation was imputed for those missing wave 2 completely. Individuals with dementia at wave 2 (or imputed dementia at wave 2) were assumed to have developed the dementia mid way between the study waves. Incidence was calculated in all those who were observed (or imputed) to be dementia free at wave 1. Within each imputed dataset, the incidence rate by 1,000 person years was calculated in a weighted Poisson regression model adjusting for initial non-response, using previously calculated initial non-response weights^11^ in those individuals who were either known or imputed to be without dementia at baseline.

All full likelihood models were undertaken in WinBUGS using non-informative uniform priors or flat normal priors for all parameters^35^. The only difference in the models between the studies was that in CFAS I the study design of dementia being missing by design is additionally modelled at each wave^34^. Convergence was checked using Gelman Rubin^40^. Autocorrelation within the models was removed by update thinning. All models were fitted with 1,000 burn in samples, with thinning of 10, with a further 1,000 to produce an incidence rate within the studies (not adjusted by non-response). Overall 100 imputation datasets (thinned by 10) were then created to undertake the analysis adjusted for initial non-response. All estimates were calculated separately in each imputation dataset and combined with Rubin's rules^41^, with 95% CI estimates. Mean estimates are used throughout though medians were compared and found similar. Inverse probability weighted Poisson regression was used to calculate incidence of dementia by age and sex in each study in each imputed dataset. Incidence rates are presented per 1,000 person years. The effect of area, deprivation, age and sex were investigated univariately and then multivariately in the Poisson regression models (see Supplementary Table 3 for deprivation). Robustness of the conclusions to model assumptions were tested by also using Negative Binomial regression models, but the results were unchanged. Significance values of findings were taken from the regression models.

Changes in incidence over time were investigated using the imputation samples and estimating a study effect after adjustment for age, area (as study site), deprivation and sex differences between the studies. Area (and deprivation) differences in incidence rates for each study were investigated after adjusting for age and sex effects. Modelled incidence rates are presented per 1,000 person years by age and sex. These estimates are provided (adjusted for multiple imputation) as an IRR (and 95% CI). Total numbers of incidence cases occurring in England and also the UK are calculated using population estimates by age (1991 and 2011 census population data and 2015 current population estimates). The effect of area, deprivation, age and sex were investigated univariately and then multivariately in the Poisson regression models (see Supplementary Table 3 for deprivation). Area (study site) effects are presented as an IRR with Cambridgeshire as the referent, deprivation as an IRR with lowest deprivation tertile as the referent, deprivation trends across tertiles were also estimated. The estimate of change over time was calculated adjusting for age, sex, deprivation and area differences. Change is presented as an IRR using CFAS I as the reference, and a 95% CI. The study effect was also estimated within a combined full likelihood model with study included at each step of the process mentioned above. The results were consistent with the weighted estimates.

A number of sensitivity models were run to check on the impact of the missing data on the model estimates. Including further parameters within any parts of the model did not modify the dementia incidence rates seen. Modelling the missing data as missing at random within the above detailed steps was appropriate to the study data. Modifying the missing data models to include or exclude the various prediction parameters (care, adjustments, deprivation etc) had little impact on the models hence the model considered *a priori* is presented. Sensitivity to adjustment for attrition was undertaken splitting out the adjustment factors, but the incidence rates were robust to these changes, hence the model with fewer attrition parameters is presented.

Three additional sensitivity analyses were undertaken to show that the missing data model did not determine the results. In CFAS II it is possible to undertake a simple attrition and non-response weighted Poisson regression analysis due to the measurement of dementia at both times. In CFAS I the method used in the original CFAS I incidence estimation^25^ was also undertaken, though this method has been shown to be less efficient than the full likelihood method described above^37^. In addition, the small subset of individuals who had direct incidence estimates were also used for comparison. All three methods are compared in Supplementary Table 3, and were found comparable.

### Comparison with previous results

Supplementary Table 1 shows a comparison of the presented results in survivors compared to other methods of analysis. The rates in CFAS I are compared with the analysis method used in the original incidence paper for CFAS I (ref. 25), which has been shown to be less efficient that the method developed after that publication^37^. There is some fluctuation in the results as expected due to the size of the samples but the results are consistent between the two, and as expected the new method has narrower CIs. An additional analysis using only those with directly measured incidence (and weighted back to the original sample) is also shown for comparison, with similar (at older ages) but very unstable estimates. The second comparison takes the full likelihood Bayesian model and imputation and compares with the usual method of analysis for incidence of a weighted Poisson regression analysis possible in CFAS II, the results are completely consistent.

## Additional information

**How to cite this article:** Matthews, F. E. *et al*. A two decade dementia incidence comparison from the Cognitive Function and Ageing Studies I and II. *Nat. Commun.* 7:11398 doi: 10.1038/ncomms11398 (2016).

## Supplementary Material

Supplementary Information Supplementary Tables 1-3

## Figures and Tables

**Table 1:** Incidence rates per 1,000 person years by age and sex (95% confidence intervals (CI)).

	CFAS I		CFAS II	
	Rate	95% CI	Rate	95% CI
*Men (age years)*				
65–69	10.3	(5.4–19.4)	5.0	(2.5–10.2)
70–74	12.9	(7.0–23.8)	8.7	(5.1–15.1)
75–79	20.4	(7.7–54.1)	16.7	(10.5–26.4)
80–84	42.4	(25.2–71.3)	24.8	(15.5–39.8)
85+	71.5	(36.5–140.2)	38.0	(22.5–64.2)
				
*Women (age years)*				
65–69	6.3	(3.0–13.3)	4.6	(2.2–9.6)
70–74	7.4	(3.8–14.8)	6.4	(3.3–12.3)
75–79	17.6	(10.8–28.7)	16.1	(10.0–25.8)
80–84	35.6	(24.0–52.8)	39.6	(28.8–54.5)
85+	59.5	(40.7–87.1)	55.3	(39.0–78.3)

**Table 2:** Incidence rates per 1,000 person years (95% confidence intervals (CI)) and unadjusted incidence rate ratios (IRR), by study, area and deprivation tertile.

	CFAS I			CFAS II		
	Rate	95% CI	IRR (95% CI)	Rate	95% CI	IRR (95% CI)
Overall rate	20.1	(16.8–24.0)		17.7	(15.2–20.6)	
						
Cambridgeshire	19.1	(14.0–26.2)	1	16.0	(12.2–21.0)	1
Newcastle	16.7	(11.7–23.6)	1.0 (0.5–1.6)	20.6	(16.1–26.4)	1.3 (0.9–1.9)
Nottingham	24.8	(19.0–32.3)	1.3 (0.8–2.0)	16.4	(12.6–21.4)	1.0 (0.7–1.5)
						
Least deprived	19.7	(14.6–26.5)	1	14.0	(10.7–18.3)	1
Middle	21.0	(15.9–27.8)	1.1 (0.7–1.6)	18.7	(14.7–23.8)	1.3 (0.9–1.9)
Most deprived	18.8	(14.0–25.2)	1.0 (0.6–1.5)	20.6	(15.8–26.8)	1.5 (1.0–2.2)
