# Supplementary material for: A two decade dementia incidence comparison from the Cognitive Function and Ageing Studies I and II
Source: Nat Commun. 2016 Apr 19;7:11398. doi: 10.1038/ncomms11398 (PMC4838896; doi:10.1038/ncomms11398)
Supplement: Supplementary Information — Supplementary Tables 1-3 [file ncomms11398-s1.pdf]

Supplementary table 1: Total numbers, proportion died and numbers with known dementia status by study

	CFAS I					CFAS II				
	Baseline interview at wave 1	Interviewed at wave 2	All dementia / Known status	Incident cases / Free of dementia at baseline	Died (%)	Baseline interview at wave 1	Interviewed at wave 2	All cases	Incident cases / Free of dementia at baseline	Died (%)
Area										
Cambridgeshire	2601	1720	114 / 629	30 / 247	10.6	2558	1755	87 / 1755	53 / 1721	6.8
Newcastle	2522	1705	106 / 551	25 / 251	10.1	2582	1751	109 / 1732	64 / 1687	8.7
Nottingham	2512	1731	147 / 619	31 / 251	11.5	2622	1782	79 / 1782	56 / 1759	9.3
Men										
65-69	915	696	15 / 203	5 / 101	6.2	968	738	8 / 738	8 / 738	3.0
70-74	780	562	18 / 155	7 / 83	8.6	902	673	23 / 673	13 / 663	5.2
75-79	696	466	22 / 134	3 / 48	15.8	758	531	29 / 531	18 / 520	6.9
80-84	449	271	27 / 97	6 / 29	20.7	542	347	33 / 347	17 / 331	13.7
85+	205	110	28 / 55	5 / 18	28.8	364	193	22 / 186	14 / 178	25.3
Women										
65-69	1066	778	11 / 218	6 / 113	2.4	971	741	9 / 741	7 / 739	2.4
70-74	996	693	18 / 223	7 / 112	4.3	971	696	12 / 696	9 / 693	2.9
75-79	1029	718	42 / 239	13 / 96	8.3	866	591	32 / 591	18 / 577	5.1
80-84	859	526	93 / 257	14 / 83	14.9	736	456	50 / 456	37 / 443	11.7
85+	640	336	93 / 218	20 / 66	23.6	684	322	57 / 310	32 / 285	24.6
Total										
65-69	1981	1474	26 / 421	11 / 214	4.2	1939	1479	17 / 1479	15 / 1477	2.7
70-74	1776	1255	36 / 378	14 / 195	6.2	1873	1369	35 / 1369	22 / 1356	4.0
75-79	1725	1184	64 / 373	16 / 144	11.3	1624	1122	61 / 1122	36 / 1097	5.9
80-84	1308	797	120 / 354	20 / 112	16.9	1278	803	83 / 803	54 / 774	12.5
85+	845	446	121 / 273	25 / 84	24.9	1048	515	79 / 496	46 / 463	24.8

Supplementary Table 2: Impact of adjustment on deprivation analysis

**CFAS I**	**Not adjusted**	**Adjusted for age and sex**	**Adjusted for age, sex and area**
**Least deprived**	1	1	1
**Middle**	1.1 (0.7-1.6)	1.0 (0.7-1.5)	1.0 (0.6-1.4)
**Most deprived**	1.0 (0.6-1.5)	0.9 (0.6-1.4)	0.8 (0.5-1.3)
**Trend**	1.0 (0.8-1.2) p=0.83	1.0 (0.8-1.2) p=0.79	0.92 (0.7-1.1) p=0.44
**CFAS II**			
**Least deprived**	1	1	1
**Middle**	1.3 (0.9-1.9)	1.3 (0.9-1.8)	1.3 (0.9-1.8)
**Most deprived**	1.5 (1.0-2.2)	1.3 (0.9-2.0)	1.3 (0.8-1.9)
**Trend**	1.21 (1.0-1.5) p=0.05	1.15 (0.9-1.4) p=0.16	1.12 (0.9-1.4) p=0.26

Supplementary Table 3: Comparison of methods (modelled incidence rate)

	CFAS I					
	Full likelihood		Pseudolikelihood		Direct incidence only	
Age						
65-69	8.2	(4.9-13.5)	8.4	(4.4-14.8)	11.86	(4.4-41.5)
70-74	9.8	(6.1-15.8)	8.9	(4.5-16.2)	17.35	(7.6-48.0)
75-79	18.3	(11.7-28.7)	17.1	(10.0-25.5)	18.58	(10.8-33.9)
80-84	38.0	(27.4-52.8)	31.6	(21.5-45.7)	35.58	(20.9-63.2)
85+	62.6	(44.7-87.7)	65.9	(45.3-91.3)	77.93	(46.4-133.4)

	CFAS II			
	Full likelihood		Poisson model – weighted	
Age				
65-69	4.8	(2.9-8.1)	4.9	(2.4-7.5)
70-74	7.5	(4.9-11.5)	7.6	(4.4-10.8)
75-79	16.4	(11.7-22.9)	16.9	(11.3-22.5)
80-84	33.6	(25.8-43.8)	33.3	(24.3-42.3)
85+	49.4	(36.6-66.5)	51.5	(36.3-66.7)

Rates per 1000 person years and 95% confidence intervals
